# Patterns of Improvement in Resection of Hepatocellular Carcinoma in Cirrhotic Patients: Results of a Non Drainage Policy

**DOI:** 10.1155/1989/31824

**Published:** 1989

**Authors:** C. Smadja, L. Berthoux, J. L. Meakins, D. Franco

**Affiliations:** 1 Groupe de Recherche sur la Chirurgie du Foie et de l'Hypertension Portale Hôpital Paul Brousse Villejuif France; 2 Hôpital Bicêtre Le Kremlin Bicêtre France; 3 Hôpital Louise Michel Evry France; 4 Hôpital Paul Brousse Villejuif cédex 94804 France

## Abstract

A prolonged ascitic leak through abdominal drains is a source of postoperative complications and of prolonged postoperative hospital stay after liver resection for hepatocellular carcinoma (HCC) in cirrhotic patients. Therefore we elected to abstain from routine abdominal drainage in the last 14 resections in cirrhotic livers. A significantly smaller number of patients had postoperative complications following liver resections without drainage (7%) than historical controls with abdominal drainage (59%, p < 0.01). The number of complications related to ascites was significantly greater in patients with abdominal drainage (76%) than without (0%, p < 0.001). Postoperative hospital stay was also significantly longer following resections with abdominal drainage (19 ± 4 days) than in patients without (12 ± 1 days, p < 0.01). The long postoperative hospital stay in patients with abdominal drainage was related to ascitic discharge for a mean period of 13 ± 10 days. No clinically significant accumulation of ascites was noted in patients without drainage. A more frequent utilization of hepatic vascular inflow occlusion did not account for the better results in the group of patients without drainage. These results suggest that routine abdominal drainage should not be used following liver resection for HCC in cirrhotic patients. This appears to be another of the technical details improving postoperative results in these patients.


*HPB Surgery* 1989, Vol. 1, pp. 141–147
Reprints available directly from the publisher
Photocopying permitted by license only

© 1989 Harwood Academic Publishers GmbH
Printed in Great Britain

### PATTERNS OF IMPROVEMENT IN RESECTION OF HEPATOCELLULAR CARCINOMA IN CIRRHOTIC PATIENTS: RESULTS OF A NON DRAINAGE POLICY

C. SMADJA[1], L. BERTHOUX[2], J.L. MEAKINS[2] and D. FRANCO[3]*

*Groupe de Recherche sur la Chirurgie du Foie et de l'Hypertension Portale,[1] – Hôpital Bicêtre, Le Kremlin Bicêtre,[2] – Hôpital Louise Michel, Evry, and[3] – Hôpital Paul Brousse, Villejuif, France*

*(Received 18 July 1988)*

A prolonged ascitic leak through abdominal drains is a source of postoperative complications and of prolonged postoperative hospital stay after liver resection for hepatocellular carcinoma (HCC) in cirrhotic patients. Therefore we elected to abstain from routine abdominal drainage in the last 14 resections in cirrhotic livers. A significantly smaller number of patients had postoperative complications following liver resections without drainage (7%) than historical controls with abdominal drainage (59%, $p < 0.01$). The number of complications related to ascites was significantly greater in patients with abdominal drainage (76%) than without (0%, $p < 0.001$). Postoperative hospital stay was also significantly longer following resections with abdominal drainage (19 ± 4 days) than in patients without (12 ± 1 days, $p < 0.01$). The long postoperative hospital stay in patients with abdominal drainage was related to ascitic discharge for a mean period of 13 ± 10 days. No clinically significant accumulation of ascites was noted in patients without drainage. A more frequent utilization of hepatic vascular inflow occlusion did not account for the better results in the group of patients without drainage. These results suggest that routine abdominal drainage should not be used following liver resection for HCC in cirrhotic patients. This appears to be another of the technical details improving postoperative results in these patients.

KEY WORDS: Liver cirrhosis, liver resection, hepato-cellular, carcinoma, surgical drainage.

### INTRODUCTION

Drainage is considered "de rigeur" by most surgeons following hepatic resection. A significant proportion of post operative complications after resection of hepatocellular carcinoma (HCC) in cirrhotic patients results from ascites formation and leakage through or around abdominal drains[1-6]. This requires intensive supportive medical therapy, prolongs the postoperative hospital stay, and may lead to infection and death.

Our experience has been similar and avoiding complications related to an ascitic leak has become a prerequisite with increasing surgical experience[7]. Surprisingly, this has not been clearly emphasized by others and no simple solution to this critical problem was apparent in the large Eastern experience[8-12]. Since June 1986, because of complications secondary to postoperative drainage of ascites, we have elected to abstain from abdominal drainage after liver resection in cirrhotic patients. The aim of this paper is to evaluate the results of this non drainage policy.

* Address correspondence to: D. Franco, Hôpital Paul Brousse, 94804 Villejuif Cédex, France.

### PATIENTS AND METHODS

From May 1981 to September 1987, 28 cirrhotic patients underwent 32 hepatic resections for HCC. Two patients had two resections at intervals of six months and one year, and one patient had three, separated by 2 and 3 years. One patient who died from bleeding esophageal varices on the 5th postoperative day after segmental resection could not be evaluated and was not taken into consideration for the present study. Therefore, 27 patients with 31 liver resections were included.

There were 25 males and 2 females with a mean age of 59 years (range: 39 to 76 years). Cirrhosis was alcoholic in 21 patients, post-necrotic in 3 patients, complicating hemochromatosis in 2 patients and cryptogenic in 1 patient. The severity of liver disease at the time of resection was assessed according to Pugh's score[13]. At time of resection 26 patients were in Pugh's class A, 4 in class B and one in class C.

In all patients the technique of liver resection was the following: transection of liver parenchyma by gentle crushing with a Kelly clamp, isolation of bilio-vascular pedicles and division after ligation with resorbable sutures or by resorbable clips. The last eighteen resections were performed under temporary hepatic vascular inflow occlusion by a Pringle maneuver. The type of hepatic resection according to Couinaud's classification[14] is indicated in Table 1. There were seven major hepatectomies, 17 segmentectomies and 7 non anatomic resections. Sodium and water restriction was applied to all patients during anesthesia and throughout the postoperative period.

**Table 1** Type of 31 hepatic resections performed in 27 cirrhotic patients[a].

| | Hepatectomies with abdominal drainage (17 resections) | Hepatectomies without abdominal drainage (14 resections) |
|---|---|---|
| Right hepatectomy | 4 | 1 |
| Left hepatectomy | 1 | 1 |
| Trisegmentectomy | 1[b] | 0 |
| Bisegmentectomy | 2[c] | 3[d] |
| Segmentectomy | 7[e] | 4[f] |
| Non anatomic resection | 2[g] | 5[h] |

a– Three patients underwent more than one resection (2 had 2 resections, 1 had 3 resections).
b– Resection of segments V, VI, VII.
c– Resection of segments II-III (1 patient) and V-VI (1 patient).
d– Resection of segments V-VI (1 patient) and IV-V (2 patients).
e– Resection of segment V (3 patients), VII (1 patient) and VIII (3 patients).
f– Resection of segment IV (1 patient), V (1 patient), VII (1 patient) and VIII (1 patient).
g– Non anatomic resection of segments V, VIII (1 patient) and III (1 patient).
h– Non anatomic resection of segments IV (1 patient), VI (1 patient), VII (1 patient), IV-V (1 patient) and IV, V, VIII (1 patient).

There were two groups according to the use of abdominal drainage. The first group, historical controls, consisted of 17 hepatic resections performed before June 1986, when abdominal drainage was routine, using one or two 30 F tubular drains through separate stab wounds. Abdominal fluid was collected in sterile bottles. No suction was applied. The second group consisted of the 14 consecutive hepatic resections performed since June 1986, without abdominal drainage. Mean age,

**Table 2** Clinical preoperative status and operative data during 31 liver resections in 27 cirrhotic patients.

| | Hepatectomies with abdominal drainage (17 resections) | Hepatectomies without abdominal drainage (14 resections) |
|---|---|---|
| Mean age (years) | 59 (range : 45–72) | 56 (range : 39–76) |
| Number of alcoholic cirrhosis[a] | 11 (65%) | 10 (71%) |
| Number of Pugh A patients | 13 (76%) | 10 (71%) |
| Number of major hepatic resections | 5 (29%) | 2 (14%) |
| Hepatic vascular inflow occlusion | 6 (35%) | 12 (86%)[b] |
| Mean transfusions of packed red cells (units) | 5.6 ± 0.6 (range : 0–9) | 2.3 ± 0.5 (range : 0–5)[c] |

a: Three patients underwent more than one resection (2 had 2 resections and 1 had 3 resections).
b: $p < 0.01$.
c: $p < 0.001$.

percentage of alcoholic cirrhosis, severity of liver disease and the number of major hepatic resections were not significantly different in the two groups (Table 2). There were significantly more patients with hepatic vascular inflow occlusion ($p < 0.01$) and significantly less transfusions ($p < 0.001$) in the group without drainage (Table 2).

All postoperative complications were carefully noted. In patients with a drain, ascites was considered a complication when drainage was continued for over 10 days (ascitic leak) and/or when the abundance of ascites leak led to intensive care and/or microorganisms were cultured from the ascites flowing through the drain. In patients without drainage, ascites was considered as a complication when there was accumulation of abdominal fluid requiring diuretics and/or paracentesis.

All results are presented as means ± SE. Significance of difference was assessed by the Student's t test and the Chi-Square test.

### RESULTS

### *Postoperative Hospital Stay*

Postoperative hospital stay was significantly shorter in patients without abdominal drainage (12 ± 1 days) compared to patients with drainage (19 ± 4 days; $p < 0.01$) in whom drainage was maintained for a mean period of 13 ± 10 days.

### *Postoperative Complications*

A total number of 17 postoperative complications (55%) were recorded in 11 patients (35%) following 31 liver resections (Table 3). A significantly greater number of patients had one or more complications after liver resection with abdominal drainage (59%) than without abdominal drainage (7%, $p < 0.01$). Complications related to ascites were signficantly more frequent after resection with abdominal drainage (76%) than without abdominal drainage (0%. $p < 0.001$). Although there were more other abdominal complications in the former group than in the latter, the difference was not significant. One patient in the group of resections with abdominal drainage needed reoperation to evacuate a subphrenic hematoma.

**Table 3** Postoperative complications following 31 liver resections in cirrhotic patients.

| Nature of complications | Liver resections with drainage (17) | | Liver resections without drainage (14) | |
|---|---|---|---|---|
| | Number of complications | Number of resections with at least one complication | Number of complications | Number of resections with at least one complication |
| Complications resulting from ascites: | 13 | 7 | 0[a] | 0 |
| • Prolonged drainage | 6 | | | |
| • Intensive care | 3 | | | |
| • Infection | 4 | | | |
| Other abdominal complications: | 2 | 2 | 0 | 0 |
| • External biliary fistula | 1 | | | |
| • Subphrenic hematoma | 1 | | | |
| Right pleural effusion | 1 | 1 | 1 | 1 |
| Total number of complications | 16 | 10 | 1[b] | 1 |

a and b: significantly different than with abdominal drainage ($p < 0.001$ and $p < 0.01$) following resections.

No accumulation of ascites requiring diuretics and/or paracentesis was observed in patients without drainage.

### *Influence of Hepatic Vascular Inflow Occlusion and of Operative Bleeding on Results*

In the group with drainage, the patients having hepatic vascular inflow occlusion experienced 6 out of the 9 abdominal complications observed. Blood transfusion requirements were similar in patients with postoperative complications (5.7 ± 1.8 units of packed red cells) and without complications (5.5 ± 1.6 units of packed red cells, N.S.). These data suggest that the better results observed in patients without drainage as compared to patients with drainage could not be ascribed to the greater number of patients with hepatic vascular inflow occlusion and the lesser amount of intraoperative bleeding.

### DISCUSSION

Abdominal drainage has so far been "de rigueur" following liver resection[15,16]. This has been motivated by the high risk of postoperative complications and in particular bleeding, bile leakage and subphrenic abscesses.

Ascites formation occurs in two thirds of cirrhotic patients after abdominal surgery[17]. It is a source of major complications resulting from prolonged and abundant drainage of fluid and proteins and from infection[18]. Formation and drainage of ascites is an important cause of postoperative mortality in patients with cirrhosis and abdominal surgery[19]. This is also the case after resection of HCC in these patients[1-6]. Our abdominal surgery experience in patients with cirrhosis and ascites has led us to reassess our use of abdominal drainage. It has been successively abandonned following portacaval shunt[20], Sugiura's operation[21] and intestinal surgery in cirrhosis[22] with a low rate of abdominal complications. Our high rate of postoperative complications related to draining ascites after resection of HCC in patients with cirrhosis has now led us to stop routine drainage in these patients.

This is not a controlled study and the groups are not exactly comparable, one being a historical control and the second has had the technical benefit of greater operative experience. It is, however, noteworthy that after liver resection cirrhotic patients without drainage had no complications related to ascites and had a significantly shorter, on average 12 days, postoperative hospital stay. It is interesting to note that in one patient abdominal drainage did not prevent a subphrenic hematoma. Postoperative accumulation of ascites was never clinically significant in patients without drainage and diuretics or paracentesis were not necessary in any patient in this group.

Since patients without drainage also had more frequent intraoperative clamping of the hepatic pedicle ($p < 0.01$) and consequently less operative bleeding and blood transfusion, the question arose whether both features could account for the better results observed. In fact, there were more complications in patients with drainage and temporary clamping of the portal pedicle. There was no relationship between blood transfusion and the onset of postoperative complications.

Resection of HCC in cirrhosis can be done with low operative mortality[3,7,11]. Technical details are of great importance in achieving such results, in particular adapting the size of resection to the size of the tumor and decreasing operative bleeding. Avoiding abdominal drainage appears to be another of the technical details which have permitted improvement in results. The good results from not draining hepatic resections in cirrhotic patients, whose liver is more fragile and surgically difficult, suggests that resection of the normal liver can be done without drainage and should always be considered except when a specific technical difficulty enhances the risk of postoperative bleeding or bile leakage.

### *References*

1. Belghiti, J., Menu, Y., Cherqui, D., Nahum, H., Fekete, F. Traitement chirurgical des carcinomes hépatocellulaires sur cirrhose. Intérêt de l'échotomographie peropératoire. *Gastroentérol. Clin. Biol.* 1986; **10**: 244–7.
2. Bismuth, H., Houssin, D., Ornowski, J., Meriggi, F. Liver resections in cirrhotic patients: a western experience. *Wld. J. Surg.* 1986; **10**: 311–7.
3. Hasegawa, H., Yamazaki, S., Makuuchi, M., Elias, D. Hépatectomies pour hépatocarcinomes sur foie cirrhotique: Schémas décisionnels et principes de réanimation péri-opératoire. Expérience de 204 cas. *J. Chir.* (Paris) 1987; **124:** 425–31.
4. Lin, T.Y., Lee, S.C., Chen, K.M., Chen, C.C. Role of surgery in the treatment of primary carcinoma of the liver: a 31-year experience. *Br. J. Surg* 1987; **74**: 839–42.
5. Nagasue, N., Yukaya, H., Ogawa, Y., Chang, Y.C., Kohno, H., Nakamura, T. Concurrent treatment of hepatocellular carcinoma and esophageal varices by hepatic resection and distal splenorenal shunt. *Arch. Surg.* 1988; **123**: 509–3.
6. Gozzetti, G., Mazziotti, A., Cavallari, A., Bellusci, R., Bolondi, L., Grigioni, W., Bragaglia, R., Grazi, G.L., De Raffele, E. Clinical experience with hepatic resections for hepatocellular carcinoma in patients with cirrhosis. *Surg. Gyn. Obstet.* 1988; **166**: 503–10.
7. Smadja, C., Berthoux, L., Kahwaji, F., Kemeny, F., Grange, D., Franco, D. Résections des carcinomes hépatocellulaires sur cirrhose: résultats d'une étude prospective de 28 résections. Gastroentérol. *Clin. Biol.* 1988; **12**: 93–8.
8. Okuda, K., Ohtsuki, T., Obata, H., Tomimatsu, M., Okazaki, N., Hasegawa, H., Nakajima, Y., Ohnishi, K. Natural history of hepatocellular carcinoma and prognosis in relation to treatment. Study of 850 patients. *Cancer* 1985; **56**: 918–28.
9. Makuuchi, M., Hasegawa, H., Yamazaki, S. Ultrasonically guided subsegmentectomy. *Surg. Gynecol. Obstet.* 1985; **161**: 356–60.
10. Lee, C.S., Sung, J.L., Hwang, L.Y., Sheu, J.C., Chen, D.S., Lin, T.Y., Beasley, R.B. Surgical treatment of 109 patients with symptomatic and asymptomatic hepatocellular carcinoma. *Surgery* 1986; **99**: 481–90.
11. Nagasue, N., Yukaya, H., Ogawa, Y., Sasaki, Y., Chang, Y.C., Niimi, K. Clinical experience with 118 hepatic resections for hepatocellular carcinoma. *Surgery* 1986; **99**: 694–701.
12. Nagao, T., Inoue, S., Goto, S., Mizuta, T., Omori, Y., Kawano, N., Morioka, Y. Hepatic resection for hepatocellular carcinoma. Clinical features and long-term prognosis. *Ann. Surg.* 1987; **205**: 33–40.
13. Pugh, R.N.H., Murray-Lyon, I.M., Dawson, J.L., Pietroni, M.E., Williams, R. Transection of the esophagus for bleeding esophageal varices. *Br. J. Surg.* 1973; **60**: 646–9.
14. Couinaud, C. Le foie. Etudes anatomiques et chirurgicales. Masson, Paris, 1957.
15. Iwatsuki, S., Geis, W.P. Specific complications in surgery of the liver. *Surg. Clin. N. Amer.* 1977; **57**: 409–19.
16. Starzl, T.E., Bell, R.H., Putnam, C.W. Hepatic trisegmentectomy and other liver resections. *Surg. Gynecol. Obstet* 1975; **141**: 429–37.
17. Brown, M.W., Burk, R.F. Development of intractable ascites following upper abdominal surgery in patients with cirrhosis. *Am. J. Med.* 1986; **80**: 879–83.
18. Fekete, F., Belghiti, J., Cherqui, D., Langonnet, F., Gayet, B. Results of esophagogastrectomy for carcinoma in cirrhotic patients. A series of 23 consecutive patients. *Surg.* 7; **206**: 74–8.
19. Doberneck, R.C., Sterling, W.A., Allison, D.C. Morbidity and mortality after operation in non bleeding cirrhotic patients. *Am. J. Surg. 1983;* **146**: 306–9.
20. Franco, D. Vons, C., Traynor, O., Smadja, C. Should portal systemic shunt be re-considered in the treatment of intractable ascites in cirrhosis? *Arch. Surg.* 1988; **123**: 987–91.

21. Kahwaji, F., Smadha, C., Grange, D., Franco, D. L'intervention de Sugiura: une exclusivité japonaise? *Gastroentérol. Clin. Biol.* 1986; **10**: 633–6.
22. Le Rolland, B., Kahwaji, F., Smadja, C., Traynor, O., Grange, D., Franco, D. Management of colorectal cancer in patients with cirrhosis and a LeVeen shunt. *Int. Surg.* 1987; **72**: 93–5.

Accepted by S. Bengmark on 9 September 1988

### INVITED COMMENTARY

Although this is a problem which has been discussed for many years, it is difficult to find fault with Dr. Smadja's concepts. A low morbidity and mortality rate in a series of patients which includes the aged is indeed enviable. The infrequency of major post-operative complications is especially noteworthy. Such success is not possible without the exercise of mature judgement in each case.

The role of drainage in this situation is to provide an exit for the intra-abdominal collection of blood, bile, lymph and necrotic tissue from the cut surface of the liver. These fluids and necrotic material are the media in which an abdominal abscess can develop. On the other hand, prolonged abdominal drainage after hepatic resection can be troublesome, because it results in prolonged ascitic leak, retrograde infection and prolonged hospital stay. Therefore, I agree that drains will not be unnecessary when there is only a small bare area at the cut surface of the liver, but it is still very difficult to abstain from abdominal drainage after hepatectomy, especially in cirrhotic patients.

In our institution, all hepatectomies are drained using soft silicon tubes, (penrose type) brought out through a lateral stab wound. They are removed several days after surgery if there is no signs of bleeding. We are of the opinion that this is probably the safest approach. Moreover, important information can be obtained from drains if any mishap occurs in the abdominal cavity. Therefore the biggest problem inherent in Dr. Smadja's concepts is the lack of information that would be available from drain, if complications did occur.

We believe that of greater importance in the prevention of complications are: how long should abdominal drains be kept in place and what type of drainage open or closed, also the quality of drains is an important point to be discussed. However, it is certain that the success of Dr. Smadja's non-drainage policy after hepatectomy in patients with liver cirrhosis brings a change of thinking on conventional post-operative management of post-hepatectomy patients.

Tatsuo Yamakawa
Teikyo University Hospital at Mizonokuchi
Kawasaki 213, Japan

